# Caveolin‐1 down‐regulation is required for Wnt5a‐Frizzled 2 signalling in Ha‐Ras^V12^‐induced cell transformation

**DOI:** 10.1111/jcmm.13531

**Published:** 2018-03-04

**Authors:** Hsiu‐Kuan Lin, Hsi‐Hui Lin, Yu‐Wei Chiou, Ching‐Lung Wu, Wen‐Tai Chiu, Ming‐Jer Tang

**Affiliations:** ^1^ Department of Physiology National Cheng Kung University Tainan Taiwan; ^2^ Department of Biomedical Engineering National Cheng Kung University Tainan Taiwan

**Keywords:** caveolin‐1, cellular and mechanical transformation, exosomes, frizzled‐2, Ha‐Ras^V12^, Wnt5a

## Abstract

Caveolin‐1 (Cav1) is down‐regulated during MK4 (MDCK cells harbouring inducible *Ha‐Ras* ^*V12*^ gene) transformation by Ha‐Ras^V12^. Cav1 overexpression abrogates the Ha‐Ras^V12^‐driven transformation of MK4 cells; however, the targeted down‐regulation of Cav1 is not sufficient to mimic this transformation. Cav1‐silenced cells, including MK4/shCav1 cells and MDCK/shCav1 cells, showed an increased cell area and discontinuous junction‐related proteins staining. Cellular and mechanical transformations were completed when MDCK/shCav1 cells were treated with medium conditioned by MK4 cells treated with IPTG (MK4+I‐CM) but not with medium conditioned by MK4 cells. Nanoparticle tracking analysis showed that Ha‐Ras^V12^‐inducing MK4 cells increased exosome‐like microvesicles release compared with their normal counterparts. The cellular and mechanical transformation activities of MK4+I‐CM were abolished after heat treatment and exosome depletion and were copied by exosomes derived from MK4+I‐CM (MK4+I‐EXs). Wnt5a, a downstream product of Ha‐Ras^V12^, was markedly secreted by MK4+I‐CM and MK4+I‐EXs. Suppression of Wnt5a expression and secretion using the porcupine inhibitor C59 or Wnt5a siRNA inhibited the Ha‐Ras^V12^‐ and MK4+I‐CM‐induced transformation of MK4 cells and MDCK/shCav1 cells, respectively. Cav1 down‐regulation, either by Ha‐Ras^V12^ or targeted shRNA, increased frizzled‐2 (Fzd2) protein levels without affecting its mRNA levels, suggesting a novel role of Cav1 in negatively regulating Fzd2 expression. Additionally, silencing Cav1 facilitated the internalization of MK4+I‐EXs in MDCK cells. These data suggest that Cav1‐dependent repression of Fzd2 and exosome uptake is potentially relevant to its antitransformation activity, which hinders the activation of Ha‐Ras^V12^‐Wnt5a‐Stat3 pathway. Altogether, these results suggest that both decreasing Cav1 and increasing exosomal Wnt5a must be implemented during Ha‐Ras^V12^‐driven cell transformation.

## 1 INTRODUCTION

Caveolin‐1 (Cav1), a major component of caveolae, interacts with many signalling molecules *via* its scaffolding domain and plays an important role in signal transduction, membrane trafficking and cholesterol transport.^1^ Accumulating evidence has shown that Cav1 is reduced in tumour‐derived cells or oncogene‐transformed fibroblasts.^2^, ^3^, ^4^, ^5^, ^6^ In addition to its role as a tumour suppressor, Cav1 is also associated with the regulation of focal adhesions and integrin‐mediated actin remodelling; both mechanisms have been widely studied with respect to mechanotransduction.^7^, ^8^ Recently, we showed that cancer cells or Ha‐Ras^V12^‐overexpressing cells exhibit a different mechanical phenotype, showing cell softening and loss of stiffness sensing.^9^ Cav1 expression is down‐regulated as a consequence of Ha‐Ras^V12^‐mediated oncogenic stimulus employed using an IPTG‐inducible expression system. In NIH3T3 fibroblasts, Cav1 increases RhoA activity and ^Y397^FAK phosphorylation, which directed actin cap formation and contributes to cell elasticity and stiffness sensing. Therefore, the Ha‐Ras^V12^‐induced fibroblast‐transformed phenotype can be reversed by Cav1 re‐expression and mimicked by Cav1 silencing.^9^

Approximately 90% of human cancers occur in epithelial tissues. In the early stages of cancer, cell junctions are often disrupted.^10^ Instead of stress fibres or actin caps, circumferential actin rings are prominent in epithelial cells. These actin filaments are associated with adherens junctions and tight junctions that generate actomyosin tension,^11^ which plays a role in mechanotransduction and regulates cell stiffness.^12^, ^13^ Importantly, Cav1 recruits the E‐cadherin/β‐catenin complex to the membrane, which stabilizes the cell‐cell adhesion of normal epithelia.^14^, ^15^ Nevertheless, whether and how Cav1 down‐regulation is responsible for epithelial transformation remains unclear.

In this study, we showed that Cav1 was down‐regulated after Ha‐Ras^V12^ induction in MK4 cells. As expected, Cav1 overexpression averted the Ha‐Ras^V12^‐driven cellular and mechanical transformation of MK4 cells. However, Cav1 silencing did not elicit the cellular and mechanical transformation of MK4 or Madin‐Darby canine kidney (MDCK) cells, suggesting that multiple changes in gene expression collaboratively contribute to Ha‐Ras^V12^ transformation. A growing body of evidence suggests that exosomes transfer proteins and functional RNA, contributing to the propagation of a transformed cell phenotype.^16^, ^17^, ^18^, ^19^ Using proteomics analysis, Simpson and colleagues demonstrated that several factors carried by exosomes contributed to the Ha‐Ras^V12^‐induced epithelial‐mesenchymal transition (EMT) in MDCK cells.^20^ Thus, the impact of Ha‐Ras^V12^‐activated exosomal factors on the transformation of Cav1‐silencing MDCK cells was evaluated.

## 2 MATERIALS AND METHODS

### 2.1 Cells and culture conditions

MDCK cells, MK4 cells (MDCK transfectants harbouring pSV*lac*O*Ras* and pHβlac*I*NLS*neo* plasmids)^9^ and SiHa cells (kindly gifted from Dr. M.R. Shen, Department of Pharmacology, College of Medicine, NCKU, Taiwan) were maintained in Dulbecco's modified Eagle's medium (DMEM, Sigma‐Aldrich, St. Louis, MO, USA) supplemented with 5% calf serum (HyClone, Logan, UT, USA), 2 mmol/L L‐glutamine (Invitrogen, Carlsbad, CA, USA), penicillin and streptomycin. All cell lines were cultured at 37°C in a 5% CO_2_, humidified incubator. C59 (porcupine inhibitor) was purchased from Abcam (Cambridge, MA, USA) and dissolved in DMSO. Wnt5a was purchased from R&D systems (Minneapolis, MN, USA).

### 2.2 Plasmids, shRNA, siRNA and transfection

The Caveolin‐1‐Myc‐mRFP plasmid was kindly gifted by Dr. IR Nabi.^21^ The short hairpin RNA (shRNA) constructs shLacZ (TRCN0000072226), shCav1‐1 (TRCN0000112662) and shCav1‐2 (TRCN0000315312) were purchased from the National RNAi Core facility, Institute of Molecular Biology/Genomic Research Center, Academia Sinica, Taipei, Taiwan. Customized Stealth RNAi^™^ siRNA (Invitrogen) targeting the Canis familiaris Wnt5a transcript (Ensembl accession number ENSCAFT00000013003) was designed using the Invitrogen RNAi Designer. The siRNA sequence 5′‐GGG CAU CCA AGA GUG CCA GUA UCA A‐3′ corresponded to residues 301‐325 of Wnt5a. To generate clones stably expressing Cav1, the cells were transfected with caveolin‐1‐Myc‐mRFP plasmid using Lipofectamine 2000 (Invitrogen). After culture for 2 days, the cells were collected and sorted by flow cytometry to enrich the mRFP‐positive cells. Gene silencing *via* lentiviral shRNA vectors was performed transfection using Lipofectamine 2000 (Invitrogen) and selection using puromycin (Cayman Chemical, Ann Arbor, MI, USA). Gene silencing *via* siRNA was performed with siRNA transfection reagent (Invitrogen) according to the manufacturer's instructions.

### 2.3 Preparation and functionalization of polyacrylamide (PA) gels

Polyacrylamide (PA) gels with uniform stiffness were prepared as previously described.^9^, ^22^, ^23^ PA gels from each polymerization batch were assessed to verify consistent matrix mechanical properties using atomic force microscope. The Young's moduli of PA gels utilized in this study included soft gel (S): *E* = 0.15‐0.3 kPa, and hard gel (H): *E* = 19‐23 kPa.

### 2.4 RT‐PCR

Total RNA was extracted from cells using TRIzol reagent (Invitrogen‐Molecular Probes, Carlsbad, CA, USA) according to the manufacturer's instructions. The RNA quality was verified and reverse‐transcribed using Moloney murine leukaemia virus reverse transcriptase (Promega, Madison, WI, USA). The cDNA was subsequently used as a template for PCR using primers specific for the following genes: dog frizzled‐2 (Fzd2; forward, 5′‐TCG TGT CAC TCT TTC GCA TC‐3′; reverse, 5′‐TGG TGA GAC GCG TGT AGA AC‐3′); human Wnt5a (forward, 5′‐CTT GGT GGT CGC TAG GTA TG‐3′; reverse, 5′‐CCT TCG ATG TCG GAA TTG AT‐3′); Ras (forward, 5′‐AGG AGC GAT GAC GGA ATA TAA G‐3′; reverse, 5′‐ACG TCA TCC GAG TCC TCC AC‐3′); and β‐actin (forward, 5′‐ACC AAC TGG GAC GAT ATG GAG AAG A‐3′; reverse, 5′‐TAC GAC CAG AGG CAT ACA GGG ACA G‐3′). PCR was performed at 94°C for 5 minutes, followed by 25 cycles at 94°C for 30 seconds, 60°C for 30 seconds and 72°C for 30 seconds, with a final step at 72°C for 10 minutes. The PCR products were resolved on a 1.2% agarose gel containing ethidium bromide and visualized under a UV transilluminator.

### 2.5 Measurements of cell stiffness by atomic force microscopy

The JPK NanoWizard^®^ II AFM with BioCell (JPK Instruments, Berlin, Germany) was used as previously described.^24^ The measurements of cell stiffness were performed as previously described.^9^, ^24^

### 2.6 Immunofluorescence staining and confocal microscopy

Immunofluorescence staining was performed as previously described.^22^ The following primary antibodies were used: Cav1, β‐catenin and E‐cadherin (BD Biosciences Pharmingen; San Jose, CA, USA), and claudin‐1 and ZO‐1 (Invitrogen). After extensively washing with PBS, the cells were incubated with secondary antimouse or rabbit IgG conjugated with Alexa 488 (Invitrogen‐Molecular Probes) and/or phalloidin‐TRITC (Sigma‐Aldrich) and 10 μg/mL Hoechst 33258 (Sigma‐Aldrich) for 1 hour. The imaging was performed from sequential *z*‐series scans using the FluoView™ FV1000 confocal microscope (Olympus, Tokyo, Japan) with a 60× water immersion lens, NA 1.35 (Uplsapo).

### 2.7 Western blot analyses

Western blot analysis was performed as previously described.^22^ Primary antibodies directed against the following proteins were used: Cav‐1, FAK, β‐catenin and E‐cadherin from BD Biosciences Pharmingen; Fzd2, Wnt5a, Cav‐1, β‐actin from Abcam (Cambridge, MA, USA); ^pY397^FAK and claudin‐1 from Invitrogen; ^pY705^‐STAT3, STAT3, pERK, ERK from Cell Signaling (Boston, MA, USA); Pan‐Ras from Calbiochem; α‐tubulin from Santa Cruz Biotechnology, Inc. (Santa Cruz, CA, USA); and β‐actin and Wnt5a from GeneTex (Irvine, CA, USA).

### 2.8 Transwell migration assay

Migration was evaluated using a 24‐well transwell assay (8 μm pore size polycarbonate membrane, Corning, MA, USA) as previously described.^9^ Briefly, 5 × 10^4^ cells from each clone were suspended in 300 μL of serum‐free DMEM and subsequently seeded onto the upper chamber, whereas 600 μL of DMEM containing 10% FBS and 10 μg/mL of collagen I was added to the outer side of the chamber. After culturing at 37°C and 5% CO_2_ in a humidified incubator for 6 hour, the cells on the upper surface of the membrane were removed using a cotton‐tipped swab, and the penetrated cells on the lower membrane surface were fixed using 4% paraformaldehyde and subsequently stained with crystal violet. Cell migration values were determined by counting all penetrated cells of each clone under a phase‐contrast microscope (200× magnification) and subsequently normalized to the control.

### 2.9 Evaluation of cell proliferation with Click‐iT^®^EdU

Cell proliferation was evaluated using a Click‐iT EdU Alexa Fluor 488 Imaging Kit (Invitrogen‐Molecular Probes) as previously described.^9^

### 2.10 Preparation of conditioned medium (CM) and isolation of exosomes

The cells were cultured under normal culture conditions. At approximately 90% confluence, the cells were rinsed twice with sterile PBS and changed to fresh culture medium to initiate conditioning. After 48 h of incubation, CM was collected and centrifuged at 2000 *g* to remove cells and debris. The supernatants were filtered through a 0.22‐μm filter, aliquoted and subsequently stored at −80°C until further use. Exosomes were isolated using total exosome isolation reagent (Invitrogen) according to the manufacturer's instructions. Briefly, the cell‐free CM was mixed well with 0.5 volumes of total exosome isolation reagent and incubated at 4°C overnight. Subsequently, the mixtures were centrifuged at 10 000 *g* for 1 hour at 4°C. Finally, the resulting exosomes pellets were resuspended in PBS and stored at 4°C for up to 1 week or at −20°C for long‐term storage.

### 2.11 Exosome labelling and uptake analysis

The exosomes were labelled with 1,1′‐Dioctadecyl‐3,3,3′,3′‐tetramethylindocarbocyanine perchlorate (DiI, Sigma). Briefly, the purified exosomes (1 mg/ml in PBS) were incubated with DiI (5 μg/mL) at 4°C for 20 minutes in the dark with gentle agitation. DiI‐labelled exosomes were washed twice with PBS by centrifugation at 10 000 *g* for 1 hour at 4°C. The pelleted exosomes were finally resuspended in PBS and stored at 4°C. For exosome uptake studies, subconfluent cells were incubated with DiI‐labelled exosomes (100 μg/mL) for 24 hour at 37°C. Surface‐bound exosomes were removed after extensive washing with serum‐free medium. Finally, cells were fixed with 2% paraformaldehyde at room temperature and observed using confocal microscopy. Exosomes uptake was calculated in representative cells using ImageJ software, and the results are represented as (the intensity of red pixels divided by cell area)*1000.

### 2.12 Nanoparticle tracking analysis (NTA)

The data presented in this study were generated using NanoSight LM10 (NanoSight Ltd., Minton Park, UK). Particles were automatically tracked and sized based on Brownian motion and the diffusion coefficient with NTA. Filtered PBS was used as a blank. CM from the 10 000 *g* centrifugation step was diluted 1/10 with PBS and subsequently used for NTA. Briefly, a 0.2‐0.3 mL of sample was loaded onto the sample chamber. The NTA measurement conditions were set as follows: temperature, 21°C; viscosity, 1 cP; frames per second, 30; measurement time, 90 seconds; and detection threshold, 5. The data are presented as the average and standard deviation of the three video recordings.

### 2.13 Statistical analyses

All data are expressed as the mean ± SEM of at least two independent experiments. The results were analysed *via* ANOVA and *t*‐tests by GraphPad Prism 3.0 (GraphPad Software, San Diego, CA). A value of *P* < .05 was deemed significant.

## 3 RESULTS

### 3.1 Cav1 overexpression abrogates the Ha‐Ras^V12^‐induced cellular and mechanical transformation of MDCK cells, whereas the targeted down‐regulation of Cav1 is not sufficient to mimic this transformation

**Figure 1:**
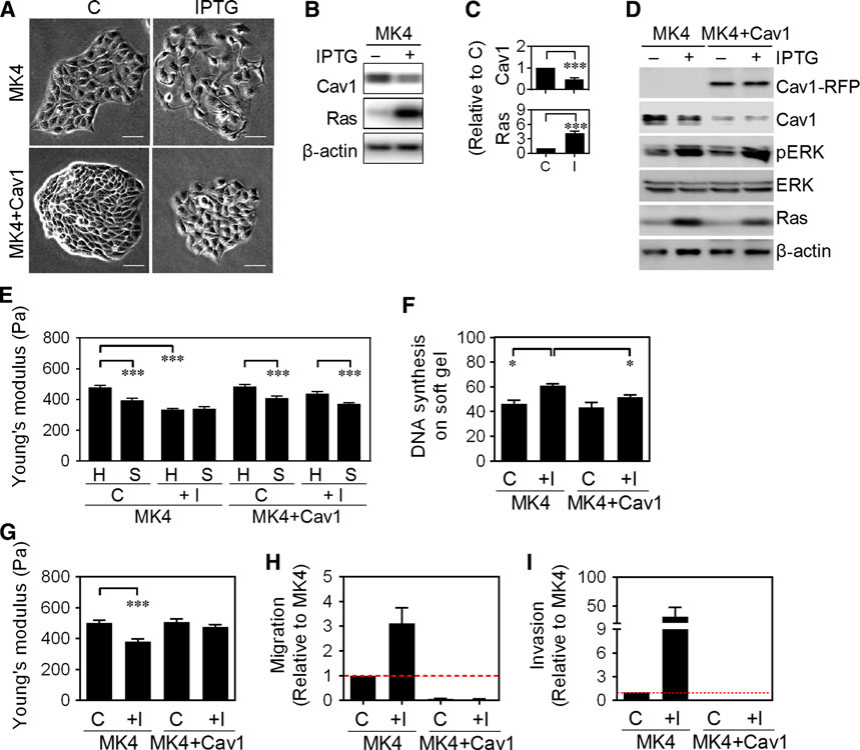
Cav1 overexpression stunts Ha‐Ras^V12^‐induced cellular and mechanical transformation. A, Representative phase‐contrast images of MK4 cells and RFP‐Cav1‐overexpressed MK4 (MK4+Cav1) cells treated with or without IPTG (5 mmol/L) for 24 h. Scale bars = 100 μm. B, Representative immunoblots for Ras and Cav1 in MK4 cells treated with or without IPTG (5 mmol/L) for 24 h. β‐actin served as an internal control. C, Quantitative results of Cav1 and Ras were from B and four other experiments (n = 5). β‐actin‐normalized data in each condition were compared with those of control. D, Representative immunoblots for Cav1‐RFP, Cav1, pERK, ERK and Ras in MK4 and MK4+Cav1 cells treated with or without IPTG (5 mmol/L) for 24 h. β‐actin served as an internal control. E, AFM indentation for cell stiffness in MK4 and MK4+Cav1 cells cultured on stiff PA gel (H) or soft PA gel (S) and treated with or without IPTG for 24 h (n = 3). F, DNA synthesis for MK4 and MK4+Cav1 cells cultured on soft PA gel and treated with or without IPTG for 24 h (n = 4). G, AFM indentation for cell stiffness in cells and treatment as described in D, (n = 3). H, Transwell migration and I, Matrigel invasion of MK4 and MK4+Cav1 cells treated with or without IPTG (n = 2). Error bars indicate means ± SEM; *P < .05, ***P < .001.

Using MDCK cells harbouring inducible Ha‐Ras^V12^ expression (MK4 cells), we showed that Ha‐Ras^V12^ induction induced cellular and mechanical transformation.^9^ Morphologically, Ha‐Ras^V12^‐overexpressing MK4 cells exhibited dramatic changes compared with typical epithelial colonies to scattered and motile single cells (Figure 1A, upper panel). Immunostaining results showed that the junction‐related proteins, including E‐cadherin, vinculin, α‐catenin, β‐catenin, γ‐catenin and ZO1, were re‐distributed from cell junctions to the cytosol without altering protein levels upon Ha‐Ras^V12^ induction (Figure S1A and B). In addition, the expression of Cav1, which co‐localized with E‐cadherin/β‐catenin/γ‐catenin in cell junctions and was suggested to regulate cell adhesion‐mediated processes,^14^ was down‐regulated (Figure 1B and C). To understand whether Cav1 down‐regulation is a prerequisite in Ha‐Ras^V12^‐induced cell scattering and transformation, Cav1‐RFP was overexpressed in MK4 cells (MK4+Cav1 cells) (Figure 1D). MK4+Cav1 cells displayed a more compact colony morphology than MK4 cells (Figure 1A, lower panel) and maintained cortical actin and junction‐related proteins at the junctions despite of Ha‐Ras^V12^ induction (Figure S1C‐E). MK4 cells altered cell stiffness on matrices of varying stiffness (Figure 1E) and were sensitive to soft matrix‐induced growth arrest (Figure 1F).^9^ Induction of Ha‐Ras^V12^ by IPTG caused cell softening (Figure 1G), loss of stiffness sensing (Figure 1E) and increased proliferation on soft gel (Figure 1F). Overexpression of Cav1 did not change these mechanical phenotypes of MK4 cells (Figure 1E‐G). However, overexpression of Cav1 conferred resistance to Ha‐Ras^V12^‐induced loss of stiffness sensing (Figure 1E), increased proliferation on soft matrix (Figure 1F) and cell softening (Figure 1G). Additionally, Cav1 overexpression inhibited the migration and invasion in MK4 cells, with or without induction of Ha‐Ras^V12^ (Figure 1H and I).

To further underscore whether the down‐regulation of Cav1 is sufficient to mediate cellular and mechanical transformation, we used shRNA to deplete Cav1 in MK4 cells (MK4/shCav1 cells, clones #1 and #2) and MDCK cells (MDCK/shCav1 cells, clones #1 and #2) (Figure S2A and B). The targeted down‐regulation of Cav1 did not change cell elasticity (Figure S2C and D) or migration (Figure S2E and F) in either MK4/shCav1 or MDCK/shCav1 cells. Both Cav1‐silenced epithelial cells displayed larger cell areas than their parental cells and shNC cells (Figure S2G‐J). Immunostaining results revealed that the junction‐related proteins, including E‐cadherin, β‐catenin and ZO‐1, were discontinuously stained on the membrane and evenly dispersed throughout the cytosol of Cav1‐depleted MDCK cells (Figure S3A). Altogether, the reduction in Cav1 in MDCK is necessary but not sufficient to mediate Ha‐Ras^V12^‐induced cellular and mechanical transformation, suggesting that multiple changes contribute to Ras transformation.

### 3.2 Exosomes derived from MK4 cells after Ha‐Ras^V12^ induction (MK4+I‐EXs) elicit the cellular and mechanical transformation of MDCK/shCav1 cells

**Figure 2:**
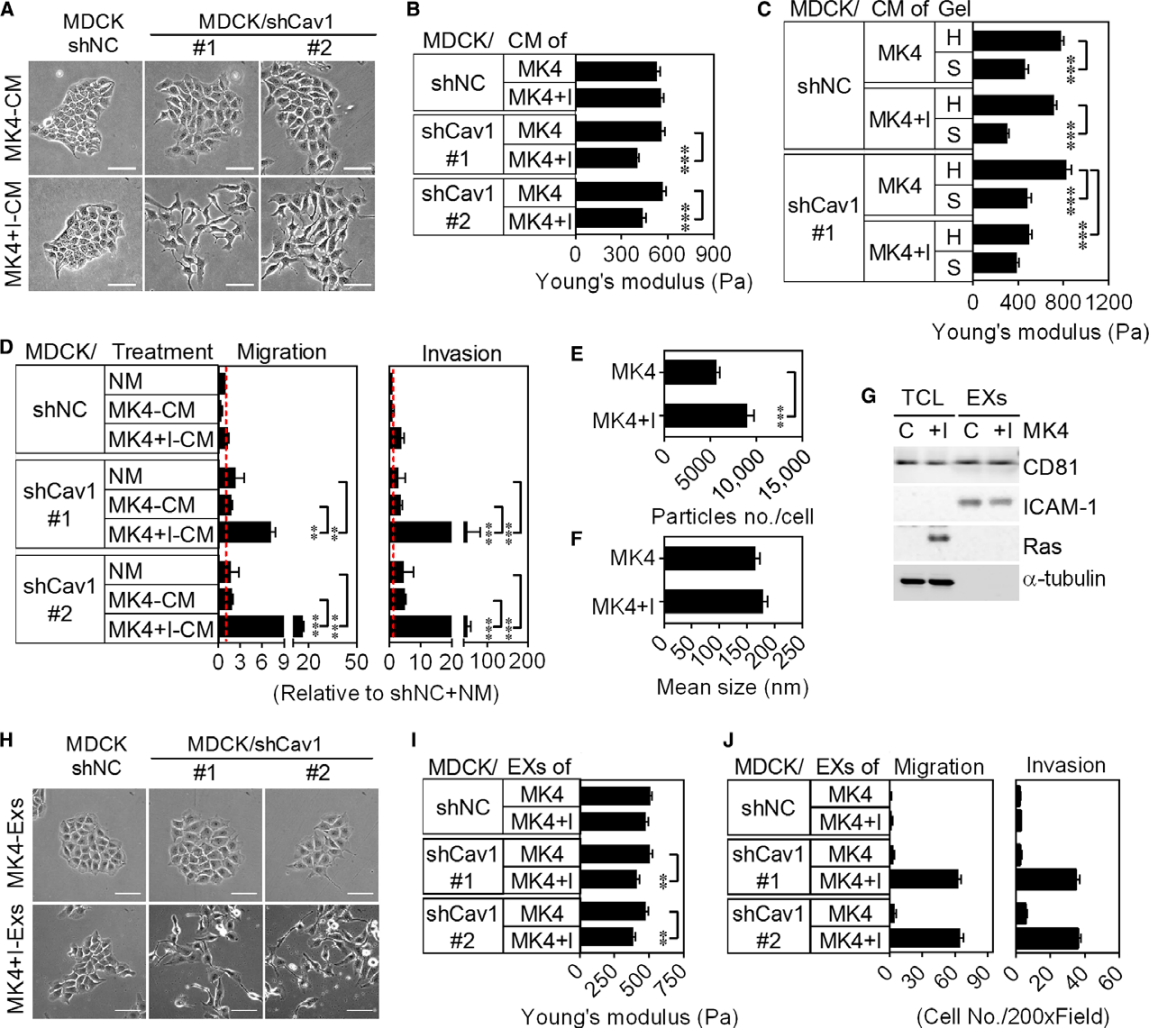
Silencing Cav1 promotes the responsiveness of MDCK/shCav1 cells to Ha‐Ras^V12^‐activated exosomes. MDCK cells were stably transfected with non‐target control shRNA (MDCK/shNC) or Cav1 shRNA (MDCK/shCav1, clones #1 and #2). A, Phase‐contrast images of the indicated cells treated with normal medium, medium conditioned by MDCK cells (MDCK‐CM), MK4 cells (MK4‐CM) or MK4 cells treated with IPTG (MK4+I‐CM). Scale bars = 100 μm. B, AFM indentation results for cell stiffness of the indicated cells treated with MK4‐CM or MK4+I‐CM for 24 h (n = 4). C, AFM indentation results for cell stiffness of the indicated cells cultured on hard (H) or soft (S) PA gels treated with MK4‐CM or MK4+I ‐CM for 24 h (n = 3). D, Transwell migration and Matrigel invasion of the indicated cells treated with MK4‐CM or MK4+I‐CM for 24 h (n = 3). E, Extracellular vesicle enumeration and F, size measurement of MK4 cells treated with or without IPTG for 24 h by nanoparticles tracing analysis (n = 5). G, Representative immunoblots for CD81, ICAM‐1 and Ras in total cell lysate (TCL) and exosomes (EXs) collected from MK4 cells treated with or without IPTG for 24 h. α‐tubulin served as a loading control. H, Phase‐contrast images of the indicated cells treated with exosomes derived from MK4 cells (MK4‐EXs) or MK4 cells treated with IPTG (MK4+I‐EXs) for 24 h. Scale bars = 100 μm. I, AFM indentation results for cell stiffness of the indicated cells treated with MK4‐EXs or MK4+I‐EXs at 300 μg/mL for 24 h (n = 3). J, Transwell migration and Matrigel invasion of the indicated cells treated with MK4‐EXs or MK4+I ‐EXs for 24 h (n = 2). Error bars indicate mean ± SEM; **P < .01, ***P < .001.

To understand whether extracellular microvesicles mediate Ras transformation, we collected medium conditioned by MDCK cells (MDCK‐CM), MK4 cells (MK4‐CM) or MK4 cells treated with IPTG (MK4+I‐CM). The colony morphology of MDCK/shNC cells was retained in all CM examined (Figure 2A). Notably, the colonic MDCK/shCav1 cells were scattered only with the administration of MK4+I‐CM (Figure 2A). Immunostaining results showed that the junction‐related proteins of MDCK/shCav1 cells disappeared and became scattered only in response to MK4+I‐CM (Figure S3B). After treatment with MK4+I‐CM, MDCK/shCav1 cells softened (Figure 2B) and failed to tune their stiffness to comply with that of the matrix to which these cells were adhered (Figure 2C). Moreover, MK4+I‐CM treatment significantly increased cell migration and invasion ability in MDCK/shCav1 cells (Figure 2D). The cellular and mechanical transformation activities of MK4+I‐CM were abolished after heat treatment (Figure S4A‐C), suggesting that some heat‐labile components were responsible for the observed MK4+I‐CM‐mediated transformation. Microvesicles, including exosomes, ranging in size from 50 to 200 nm, are secreted from almost all cells and display a wide range of biological activities. Induction of Ha‐Ras^V12^ increased exosome‐like microvesicles release from MK4 cells without changing particle size as assessed by NTA (5677 ± 384.3 particles per MK4 cell; 9039 ± 692.7 particles per MK4 cell treated with IPTG for 24 hour) (Figure 2E and F). These microvesicles were further extracted and characterized for the presence of the exosomal markers CD81 and ICAM‐1 and the absence of Ras and α‐tubulin (Figure 2G). The cell scatter‐promoting activity of MK4+I‐CM was blocked by the depletion of exosomes and revived by the replenishment of exosomes (Figure S4D). The ability of exosomes from MK4 cells (MK4‐EXs) or MK4 cells treated with IPTG (MK4+I‐EXs) to promote cell transformation was subsequently assayed. MDCK/shNC cells retained the colony morphology (Figure 2H), cell stiffness (Figure 2I), migration and invasion (Figure 2J) for all exosomes examined. Notably, MK4+I‐EXs, but not MK4‐EXs, stimulated cell scattering (Figures S4E, and 2H) and cell softening (Figure 2I) and enhanced cellular migration and invasion (Figure 2J) in only two Cav1‐silenced MDCK/shCav1 cell lines. The transformation of MK4+I‐EXs was abolished after heat treatment (Figure S4E). Collectively, we showed that neither Cav1 reduction nor MK4+I‐CM (or MK4+I‐EXs) is sufficient to mediate Ha‐Ras^V12^‐induced cellular and mechanical transformation. The combinations of Cav1 reduction and MK4+I‐CM (or MK4+I‐EXs) triggered the cellular and mechanical transformation of MDCK, confirming multiple changes in mediating Ras transformation.

### 3.3 Wnt5a is responsible for the MK4+I‐EXs‐elicited cellular and mechanical transformation of MDCK/shCav1 cells

**Figure 3:**
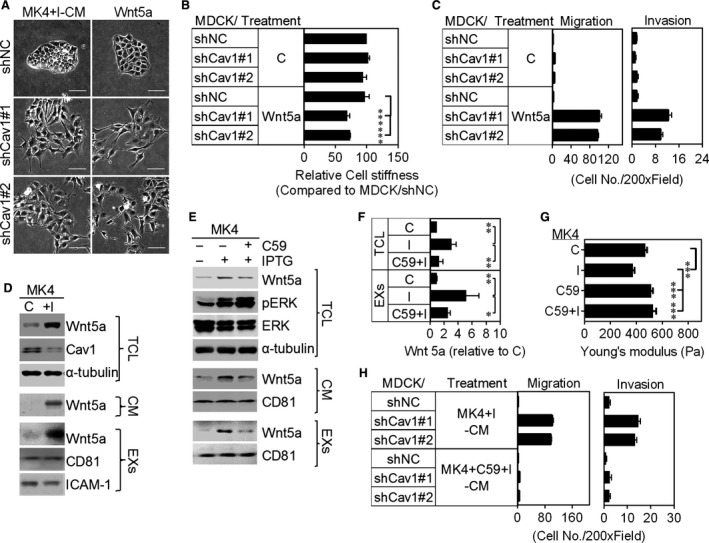
Wnt5a, which is up‐regulated upon induction of Ha‐Ras^V12^ in MK4 cells, induces cellular and mechanical transformation only in MDCK/shCav1 cells. A, Phase‐contrast images of MDCK/shNC cells and MDCK/shCav1 (clones #1 and #2) cells treated with or without Wnt5a for 24 h. Scale bars = 100 μm. B, AFM indentation results for cell stiffness of indicated cells treated with or without Wnt5a (150 ng/mL) for 24 h (n = 3). C, Transwell migration and Matrigel invasion of indicated cells treated with or without Wnt5a for 24 h (n = 2). D, Representative immunoblots for the expression of Wnt5a and Cav1 in total cell lysates, CM and exosomes derived from MK4 cells treated with or without IPTG (5 mmol/L) for 24 h. α‐tubulin served as a loading control. CD81 and ICAM‐1 served as exosomal markers. E, Representative immunoblots for Wnt5a, pERK and ERK in total cell lysate (TCL), CM and exosomes derived from MK4 cells treated with or without IPTG (5 mmol/L) in the presence or the absence of C59 (200 μmol/L) for 24 h. α‐tubulin served as a loading control. CD81 served as an exosomal marker. F, Quantitative results of Wnt5a in TCL and EXs were from D, E and two other experiments (n = 3). α‐tubulin‐normalized data in each condition were compared with those of control. G, AFM indentation results for cell stiffness of MK4 cells treated with IPTG in the presence or the absence of C59 (200 μmol/L) for 24 h (n = 3). H, Transwell migration and Matrigel invasion of the indicated cells treated with CM derived from MK4 cells treated with IPTG in the absence or presence of C59 for 24 h (MK4+I ‐CM and MK4+I+C59‐CM, respectively) (n = 2). Error bars indicate means ± SEM; **P < .01, ***P < .001.

**Figure 4:**
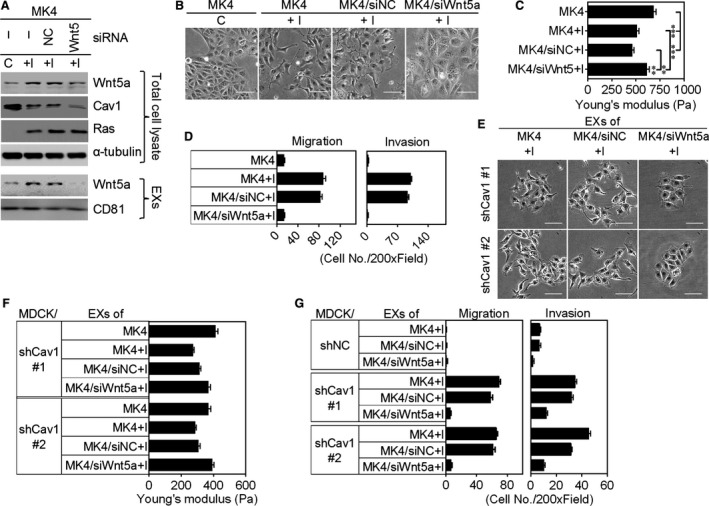
The siRNA silencing of Wnt5a abolished Ha‐Ras^V12^‐induced cellular and mechanical transformation of MK4 cells. MK4 cells, siNC‐transfected MK4 cells (MK4/siNC) or siWnt5a‐transfected MK4 cells (MK4/siWnt5a) were treated with or without IPTG (5 mmol/L) for 24 h. A, Representative immunoblots for Wnt5a, Cav1 and Ras in total cell lysates, and exosomes derived from the above‐indicated treatments. α‐tubulin and CD81 served as a loading control and exosomal marker, respectively. B, Phase‐contrast images, C, AFM indentation results for cell stiffness (n = 3) and D, Transwell migration and Matrigel invasion (n = 2) of MK4 cells treated with indicated conditions as described in A. E, Phase‐contrast images of MDCK/shCav1 cells (clones #1 and 2) treated with exosomes (EXs) derived from MK4 cells, MK4/siNC cells or MK4/siWnt5a treated with or without IPTG for 24 h. F, AFM indentation results for cell stiffness of MDCK/shCav1 cells (clones #1 and 2) treated with EXs derived from MK4 cells, MK4/siNC cells or MK4/siWnt5a treated with or without IPTG for 24 h (n = 2). G, Transwell migration and Matrigel invasion of MDCK/shNC cells and MDCK/shCav1 cells (clones #1 and #2) treated with EXs as described in F (n = 2). Scale bar = 100 μm. Error bars indicate means ± SEM; **P < .01, ***P < .001.

**Figure 5:**
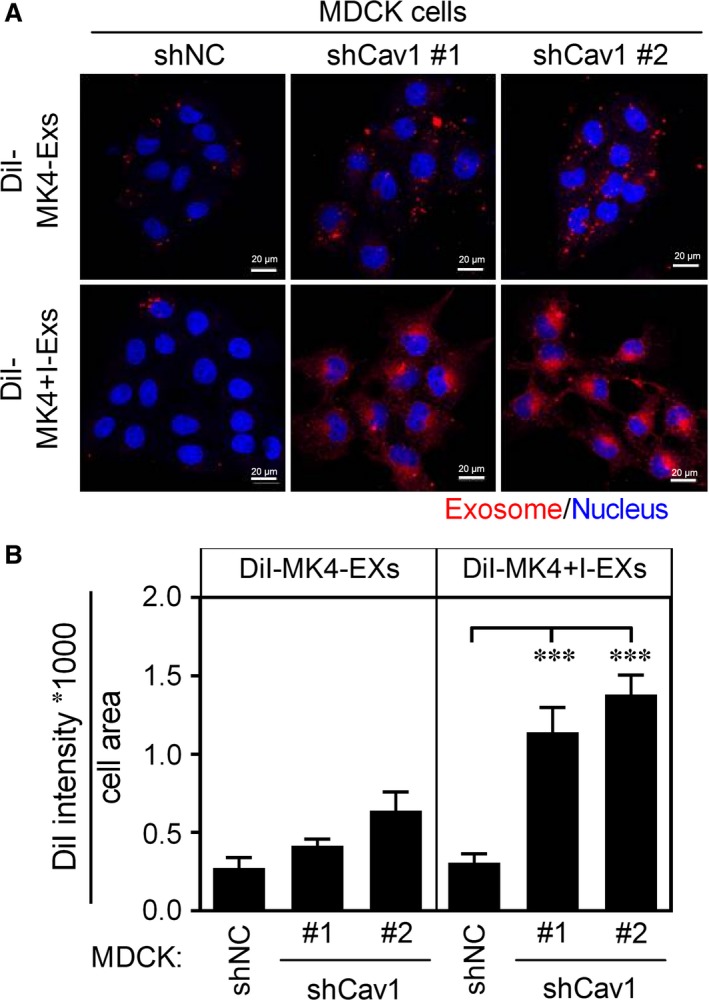
Silencing Cav1 facilitates the uptake of MK4+I‐EXs. A, MDCK/shNC cells and MDCK/shCav1 cells were incubated with DiI‐labelled exosomes (100 μg/mL) for 24 h. Cells were extensively washed with PBS to remove surface‐bound exosomes before fixation and confocal microscopy imaging. DiI‐Exosomes, red, Nucleus, blue. B, Quantification results of exosomes uptake in each condition from A. Exosomes uptake in cells were represented the intensity of red pixels * 1000 divided by cell area. Scale bar = 20 μm. Data are means ± SEM from three independent experiments; ***P < .001.

Comparative proteomics analysis of the plasma membranes of MDCK cells following oncogenic Ras/TGF β1‐mediated EMT revealed that Wnt5a was the most up‐regulated protein during Ha‐Ras^V12^‐induced EMT.^25^ The addition of Wnt5a to exosome‐depleted MK4+I‐CM rescued its cell scatter‐promoting activity (Figure S4D), indicating an important role for the Wnt5a in MK4+I‐CM‐induced cellular transformation of MDCK/shCav1. Wnt5a, similarly to MK4+I‐CM, elicited cell scattering (Figures S5A and 3A) and cell softening (Figure 3B) and enhanced cellular migration and invasion (Figure 3C) only in MDCK/shCav1 cells. We thus assessed the expression of Wnt5a in MK4 cells with or without IPTG administration. Wnt5a mRNA expression (data not shown) and protein levels (Figure 3D) were markedly increased upon Ha‐Ras^V12^ induction. Moreover, Ha‐Ras^V12^‐up‐regulated Wnt5a was secreted into the extracellular fluid through the exosomal pathway (Figure 3D). Porcupine, an O‐acyltransferase located in the endoplasmic reticulum (ER), was required for the lipidation and trafficking of Wnt5a proteins from the ER and subsequent secretion in mammalian cell culture.^26^, ^27^ The inhibition of porcupine by C59 abolished Ha‐Ras^V12^‐induced Wnt5a expression and secretion (Figures S5B, 3E and F). Consequently, C59 treatment inhibited Ha‐Ras^V12^‐induced cell scattering (Figure S5C) and cell softening (Figure 3G) in MK4 cells. Moreover, medium conditioned by MK4 cells treated with C59 and IPTG (MK4+C59+I‐CM) failed to increase the migration and invasion of MDCK/shCav1 cells compared with MK4+I‐CM (Figure 3H). To clarify the role of Wnt5a in Ha‐Ras^V12^‐induced cellular and mechanical transformation, we used specific siRNA to knockdown Wnt5a in MK4 cells. Five hundred picomoles of Wnt5a‐targeted siRNA abolished Ha‐Ras^V12^‐elevated Wnt5a protein expression and exosomal‐Wnt5a (Figures S5E and 4A). The depletion of exosomal Wnt5a prevented Ha‐Ras^V12^‐induced cell scattering (Figure 4B) and cell softening (Figure 4C) and increased the migration and invasion (Figure 4D) of MK4 cells. Notably, exosomes derived from siWnt5a‐transfected MK4 (MK4/siWnt5a) cells treated with IPTG failed to induce scattering (Figure 4E), softening (Figure 4F) and migration and invasion (Figure 4G) of MDCK/shCav1 cells. In summary, these functional data unequivocally demonstrated that Wnt5a is responsible for the MK4+I‐EXs‐induced cellular and mechanical transformation of MDCK/shCav1 cells. Considering the potency of MK4+I‐EXs associated with cellular and mechanical transformation only in MDCK/shCav1 cells, we subsequently assessed the role of Cav1 in exosome uptake. After the removal of surface‐bound exosomes, the intracellular‐labelled exosomes were imaged and quantified. The internalization of DiI‐labelled exosomes (DiI‐MK4‐EXs or Dil‐MK4+I‐EXs) was visualized using confocal fluorescence microscopy in MDCK/shNC and MDCK/shCav1 cells (Figure 5A). As shown in Figure 5B, Cav1‐expressed cells (MDCK/shNC) restricted the internalization of MK4‐EX and MK4+I‐EX. Cav1 knockout cells (MDCK/shCav1) displayed increased levels of exosome uptake, particularly in the presence of MK4+I‐EXs. These data suggested a negative regulatory role for Cav1 in exosome uptake. Additionally, the cargos of MK4+I‐EXs might contribute to the accelerated exosome uptake.

### 3.4 Cav1 suppresses Ha‐Ras^V12^/exosomal Wnt5a‐induced activation of Stat3 through the post‐translational repression of frizzled‐2 (Fzd2)

**Figure 6:**
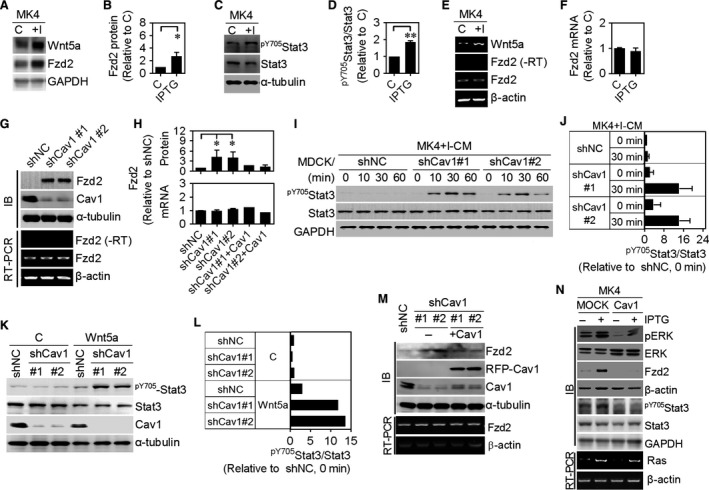
Frizzled 2 (Fzd2), inversely regulated by Cav1, mediated Ha‐Ras^V12^‐exosomal Wnt5a‐induced phosphorylation of Stat3. A, Representative immunoblots for Wnt5a and Fzd2 of MK4 cells treated with or without IPTG (5 mmol/L) for 24 h. GAPDH was used as an internal control. B, Quantitative results of Fzd2 were from A and three other experiments (n = 4). GAPDH‐normalized data in each condition was compared with those of control. C, Representative immunoblots for ^pY^ ^705^Stat3 and Stat3 of MK4 cells treated with or without IPTG (5 mmol/L) for 16 h. α‐tubulin was used as an internal control. D, Quantitative results of the ratio of ^pY^ ^705^Stat3 to Stat3 were from C and two other experiments (n = 3). α‐tubulin‐normalized data in each condition was compared with those of control. E, RT‐PCR results for Fzd2 expression of indicated cells in A. β‐actin was used as an internal control. F, Quantitative results of Fzd2 mRNA were from E and two other experiments (n = 3). β‐actin‐normalized data in each condition was compared with those of control. G, Upper panel (IB): Representative immunoblots for Fzd2 and Cav1 of MDCK/shNC cells and MDCK/shCav1 cells (clones #1 and #2). α‐tubulin was used as an internal control. Lower panel (RT‐PCR): RT‐PCR results for Fzd2 expression of indicated cells. β‐actin was used as an internal control. H, Quantitative results of Fzd2 protein and mRNA were from G and M and two other experiments (n=4). α‐tubulin‐ or β‐actin‐normalized data in each condition was compared with those of shNC cells. (**I**) Representative immunoblots for ^pY^ ^705^Stat3 and Stat3 of indicated cells treated with MK4+I‐CM for indicated times. GAPDH was used as an internal control. J, Quantitative results of the ratio of ^pY^ ^705^Stat3 to Stat3 were from I and the other experiments (n = 2). GAPDH‐normalized data in each condition was compared with those of shNC cells at time 0. K, Representative immunoblots for ^pY^ ^705^Stat3, Stat3 and Cav1 of indicated cells treated with or without Wnt5a (150 ng/mL) for 30 min. α‐tubulin was used as an internal control. L, Quantitative results of the ratio of ^pY^ ^705^Stat3 to Stat3 were from K (n = 2). M, Upper panel (IB): Western blot results for Fzd2 and Cav1 of MDCK/shNC cells, MDCK/shCav1 cells (clones #1 and #2) and Cav1‐re‐expressing MDCK/shCav1 cells. α‐tubulin was used as an internal control. Lower panel (RT‐PCR): RT‐PCR results for Fzd2 expression of indicated cells. β‐actin was used as an internal control. N, Upper panel (IB): Representative immunoblots for pERK, ERK, Fzd2, ^pY^ ^705^Stat3 and Stat3 in MK4 cells or RFP‐Cav1‐transfected MK4 cells treated with or without IPTG (5 mmol/L) for 24 h. β‐actin and GAPDH served as an internal control. Lower panel (RT‐PCR): RT‐PCR results for Ras expression of indicated cells. β‐actin was used as an internal control. For RT‐PCR, the negative control (no‐reverse transcriptase, ‐RT) reaction verifies the absence of DNA template contamination. Error bars indicate means ± SEM; *P < .05, **P < .01.

**Figure 7:**
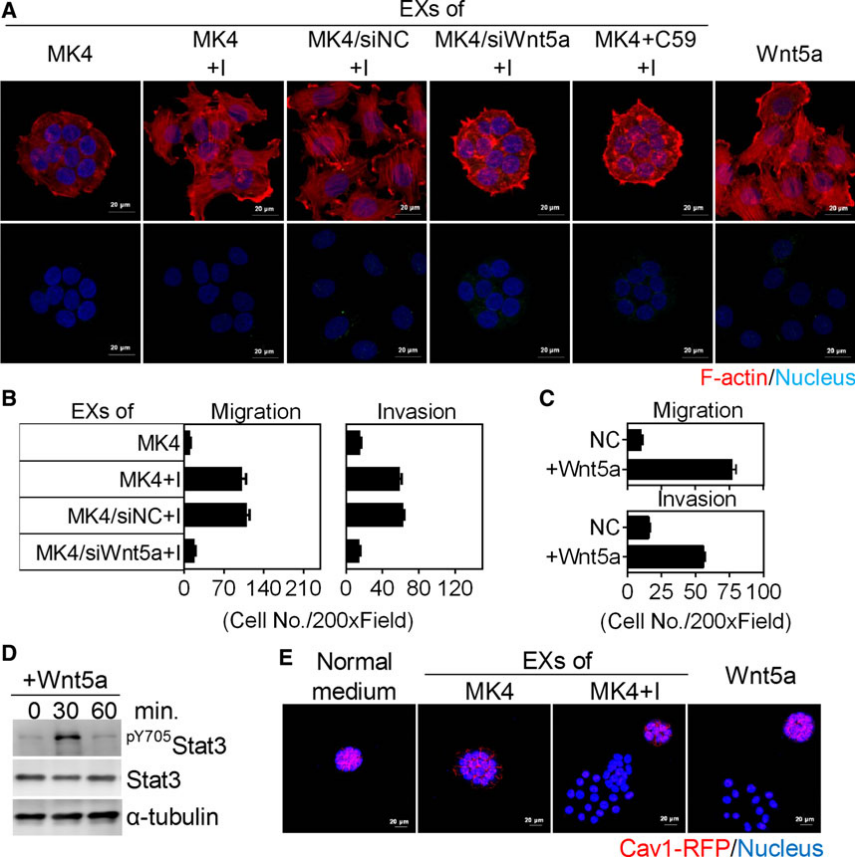
Cav1 restricted the responsiveness of SiHa cells to MK4+I‐EXs‐ or Wnt5a‐induced cell transformation. A, Representative confocal images for SiHa cells treated with exosomes (EXs) derived from MK4 cells, MK4 cells treated with IPTG, siNC‐transfected MK4 cells treated with IPTG, siWnt5a‐transfected MK4 cells treated with IPTG and MK4 cells treated with C59 and IPTG, or Wnt5a (300 ng/mL) only. F‐actin, red, Nucleus, blue. Scale bar = 20 μm. B, Transwell migration and Matrigel invasion of SiHa cells treated with EXs derived from MK4 cells, siNC‐transfected MK4 (MK4/siNC) cells and siWnt5a‐transfected MK4 (MK4/siWnt5a) cells treated with or without IPTG for 24 h (n = 2). C, Transwell migration and Matrigel invasion of SiHa cells treated with or without Wnt5a for 24 h (n = 2). D, Representative immunoblots for ^pY^ ^705^Stat3 and Stat3 in SiHa cells treated with or without Wnt5a for indicated times. α‐tubulin served as an internal control. E, Representative confocal images for Cav1‐RFP‐transfected SiHa cells treated with normal medium, EXs derived from MK4 cells treated with or without IPTG for 24 h, or Wnt5a for 24 h. Scale bar = 10 μm. Error bars indicate means ± SEM.

**Figure 8:**
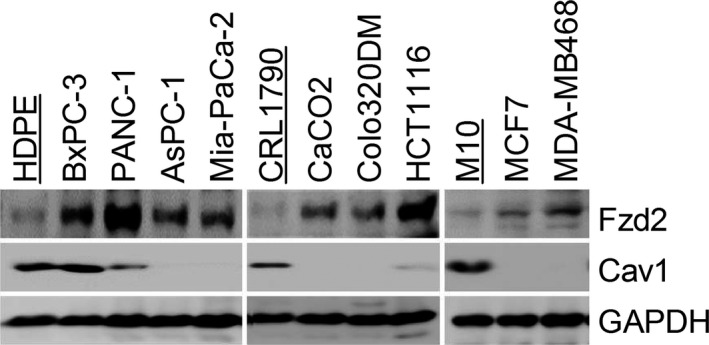
The inverse relation between protein levels of Cav1 and Fzd2 was observed in several cancer cell lines. Normal (underlined) and cancer cell lines were cultured on tissue culture dishes overnight and then harvested for Western blot analysis. GAPDH served as a loading control.

A previous study showed that Fzd2‐Stat3 signalling plays a critical role in Wnt5a‐mediated EMT and cell migration.^28^ To assess whether Fzd2‐Stat3 signalling is involved in Ha‐Ras^V12^‐Wnt5a‐induced transformation, we evaluated the Fzd2 expression and Stat3 phosphorylation in MK4 cells treated with or without IPTG. MK4 cells expressed low levels of Wnt5a and Fzd2, which were markedly increased after Ha‐Ras^V12^ induction (Figures 3D and F, 6A and B). In addition, the phosphorylation of Stat3 on Tyr^705^ was elevated, revealing that the Wnt5a‐Fzd2‐Stat3 non‐canonical pathway might be activated in Ha‐Ras^V12^‐induced transformation (Figure 6C and D). However, Fzd2 mRNA levels remained unchanged in response to Ha‐Ras^V12^ (Figure 6E and F), suggesting that Fzd2 expression is controlled through a post‐transcriptional mechanism. Silencing Cav1 increased Fzd2 protein levels without affecting its mRNA levels in MDCK/shCav1 cells (Figure 6G and H). Consequently, treatment of MDCK/shCav1 cells with MK4+I‐CM or Wnt5a increased the phosphorylation of Stat3 on Tyr^705^ (Figure 6I‐L). Re‐expression of Cav1 in MDCK/shCav1 cells decreased Fzd2 protein levels without affecting its mRNA levels (Figure 6M and H). Moreover, Cav1 overexpression in MK4 cells (Figure 1B) repressed Fzd2 protein expression without affecting its mRNA expression and prevented the Ha‐Ras^V12^/Wnt5a‐induced phosphorylation of Stat3 on Tyr^705^ (Figure 6N). Inhibition of Stat3 activity by WP1066 abolished Ha‐Ras^V12^‐ and Wnt5a‐increased migration of MK4 cells and MDCK/shCav1 cell, respectively (Figure S6). These data implicate the involvement of Stat3 phosphorylation/signalling in cell transformation in both conditions. In conclusion, Ha‐Ras^V12^‐decreased Cav1, which relieves Fzd2 expression, is imperative for Ha‐Ras^V12^/Wnt5a‐driven cellular and mechanical transformation. The above data prompted further studies of the role of Cav1 in Ha‐Ras^V12^/Wnt5a‐driven cellular and mechanical transformation. SiHa cells, expressing low levels of Cav1 compared with normal cervical epithelium,^29^ responded to MK4+I‐EXs‐ or Wnt5a‐induced cell scattering (Figure 7A) and showed increased migration and invasion (Figure 7B and C). Such effects were abolished either by siWnt5a or C59 treatment (Figure 7A and B). Treatment of SiHa cells with Wnt5a also increased the phosphorylation of Stat3 on Tyr^705^ (Figure 7D). Finally, the re‐expression of Cav1‐RFP in SiHa cells repressed MK4+I‐EX‐ or Wnt5a‐induced cell scattering (Figure 7E), confirming that Cav1 may function as a suppressor of cell transformation in cultured cells. To confirm the possible role of Cav1 in Fzd2 suppression, the levels of Fzd2 and Cav1 were evaluated in primary normal epithelial cells and well‐characterized normal and cancer cell lines derived from human pancreas, colorectal and breast tissues. Normal cells showed high expression of Cav1 with no or low expression of Fzd2. As expected, Cav1 were down‐regulated in most cancer cell lines, accompanied by the enhancement of Fzd2 (Figure 8).

## 4 DISCUSSION

In the present study, we demonstrated that Ha‐Ras^V12^ decreased Cav1 and increased exosomal Wnt5a; both are indispensable for subsequent cell transformation. These results suggest a novel role for Cav1 in inversely regulating Fzd2 protein levels, which is required for the Ha‐Ras^V12^/Wnt5a‐induced transformation of MDCK cells.

Aberrant Wnt signalling, in response to overproduction in Wnt‐secreting cells or mutations in Wnt‐receiving cells, has been implicated in many cancers.^30^ Recent studies have pointed to a critical role of exosomes for Wnt secretion and extracellular travelling.^31^, ^32^ Exosome‐bound Wnts and their signalling activities were functionally implicated during embryonic development and cancer progression.^31^, ^33^, ^34^ Here, we showed that the overexpression of Ha‐Ras^V12^ increased the synthesis and release of exosomal Wnt5a (Figure 3D‐F), which subsequently induced cellular and mechanical transformation in MK4 cells (Figures 3G and 4A‐D). Noteworthily, the level of Cav1 determines the cellular responsive to Ha‐Ras^V12^‐activated EXs, CM, or Wnt5a (Figures 5 and 6). Currently, a number of Wnt5a receptors were reported, including Fzd2, Fzd3, Fzd4, Fzd5, Fzd6, Fzd7, Fzd8, RYK, ROR2 and CD146.^35^ Results from several independent studies indicated that Fzd2 expression might drive EMT through the non‐canonical Wnt pathway in different cancer cells.^28^, ^36^, ^37^, ^38^, ^39^ Gujral et al^28^ reported that Wnt5a and its ligand Fzd2 are overexpressed in several metastatic cancer cell lines and tumours. These authors further identified a non‐canonical Fzd2‐Fyn‐Stat3 pathway that mediates Wnt5a‐induced EMT and cell migration. RT‐PCR results showed that Fzd2, but not Fzd5 and Fzd8, were highly expressed in the clones derived from MDCK cells (Figure S7). Because Ha‐Ras^V12^ induced EMT‐like morphological change in MK4 cells, we focused our research on Wnt5a‐Fzd2‐Stat3 pathway. We observed that Cav1‐reduced cells, either due to Ha‐Ras^V12^ overexpression or Cav1‐targeted shRNA, showed augmented Fzd2 protein expression (Figure 6A‐B, G‐H) and permitted the Ha‐Ras^V12^‐, MK4+I‐CM‐ or Wnt5a‐induced phosphorylation of Stat3 on Tyr^705^ (Figure 6C‐D, I‐L). Enhanced Cav1 expression not only decreased Fzd2 in MDCK/shCav1 cells (Figure 6M) but also abolished Ha‐Ras^V12^‐increased Fzd2 protein expression and Stat3 phosphorylation in MK4 cells (Figure 6N). Fzd2 protein expression thus becomes a critical gate for the autocrine and paracrine loop of Ha‐Ras^V12^‐increased exosomal Wnt5a in MK4 cells and MDCK cells, respectively. In order to identify whether Fzd2 is the receptor for Wnt5a, we have tried to deplete Fzd2 in MDCK/shCav1 to determine whether Fzd2 signalling is required for Wnt5A‐induced Stat3 phosphorylation. However, currently we have not found effective siRNA treatment for the knockdown of Fzd2 in MDCK cells (canine species). We will continue to pursue this question.

The protein levels of Cav1 inversely regulate the expression of Fzd2 protein in MDCK cells. However, the changes in Fzd2 protein were independent of changes in Fzd2 transcription, as mRNA levels of Fzd2 remained constant regardless of Cav1 levels (Figures 6E, G and M, and S7), suggesting that post‐transcriptional regulation might contribute to the observed Cav1‐elicited reduction in Fzd2. Upon cycloheximide (CHX) treatment, the protein levels of Fzd2 were increased within 4 hours only in Cav1‐expressing MK4 cells and MDCK/shNC cells (Figure S8). Inhibition of protein synthesis slightly changed the protein levels of Cav1 in each condition we tested. We propose that Cav1 facilitates the degradation of Fzd2 by suppressing the protein turnover of an unidentified molecule, which is involved in Fzd2 degradation. Inhibition of protein synthesis transiently increased Fzd2 within 4 hour, suggesting that the unidentified molecule may rapidly turnover. Therefore, in Cav1‐depleting cells, including MK4+I cell and MDCK/shCav1 clones, Fzd2 degradation is diminished and unaffected by CHX treatment within 4 hour. Cav1 has been shown to act as a scaffolding protein by binding to proteins involved in different signal transduction pathways.^40^ Accumulating evidence indicates that Cav1 regulates the degradation of signal molecules. Reduction in Cav1 expression results in the accumulation of non‐ubiquitylated and mono‐ubiquitylated Rac1, but does not affect the level of poly‐ubiquitylated Rac1, suggesting that Cav1 plays a specific role in the regulation of polyubiquitylation and subsequent degradation of Rac1.^41^ Felley‐Bosco et al^42^ reported that Cav1 is involved in the degradation of inducible nitric oxide synthase in the cytosol, but the molecular mechanism remains unclear. Chen et al^43^ showed that Cav1 cooperates with p97 to interact with Derlin‐1 and promotes the ubiquitination and degradation of cyclooxygenase‐2. Numerous studies have indicated that Cav1 mediates protein transportation from ER to the cellular membrane and regulates enzymatic activity by binding and interacting with many proteins.^44^, ^45^ Notably, co‐immunoprecipitation indicated a potential association between Cav1 and Fzd2.^46^ Whether and how Cav1 cooperates with other molecules to promote the degradation of Fzd2 or directly binds targets Fzd2 for ER‐associated degradation needs further investigation.

The importance of tumour‐derived exosomes in tumour progression cannot be overemphasized. Cancer cell‐derived exosomes promote the transformation of cells through an autocrine mechanism or through uptake by normal cells surrounding the tumour, which might confer the transformed characteristics of cancer cells upon normal recipient cells.^47^, ^48^ Bissell and Hines proposed that the microenvironment surrounding the tumour provides tumour‐suppressive signals as long as the architecture of the tissue homeostasis is essentially controlled.^49^ Although the initiation of tumours resulting from a potent oncogene is unavoidable, their progression to malignancy can and should be controllable. Overcoming the protective roles of the physiological microenvironment requires “promotion” agents, which are typically associated with aberrant repair and fibrosis. Indeed, wound healing and TGF β1 are considered highly effective promoting stimuli.^50^ Williams et al^51^ showed that Cav‐1 expression in both epithelial and stromal cells provides a protective effect against mammary hyperplasia and mammary tumorigenesis. Cav1 negatively regulates the exosome internalization in glioblastoma cells.^52^ A similar phenomenon was observed in Cav1‐expressing or silenced MDCK cells treated with Ha‐Ras^V12^‐activated exosomes (Figure 5). Exosomes carrying Wnt proteins on their surfaces were reported to activate Wnt signalling in target cells.^31^ Here, we showed that Cav1 negatively regulates the Wnt receptor Fzd2 and thereby confers a protective effect against Ha‐Ras^V12^/exosomal Wnt5a‐induced transformation in MDCK cells. Thus, the levels of Cav1 in the normal cells surrounding tumour are critical for providing tumour‐suppressive signals to constrain tumour progression. Cav1 is expressed at high levels in terminally differentiated cells and is often deregulated in cancer and fibrotic diseases.^53^ The deregulation of Cav1 *via* TGFβ, a potent fibrogenic cytokine, might disrupt tumour‐suppressive signals, thereby promoting tumour progression.

In conclusion, based on the *in vitro* evidence, we suggest that the presence of Cav1 in recipient cells blocks exosome uptake and its downstream signalling. Cav1 might play an important physiological role in the defence against tumour‐derived exosomes *via* the degradation of Fzd2, thereby suppressing Wnt5a‐driven malignant transformation or inhibition of tumour‐derived exosomes internalization through an unidentified mechanism. Although the loss of Cav1 is not sufficient to causally drive cell transformation, it is a critical step in the acquisition of the oncogene‐induced transformed phenotypes in both tumour cells and normal cells surrounding the tumour. These findings significantly advance the general understanding of exosome‐mediated tumour progression and offer potential strategies for how this pathway may be targeted through the modulation of Cav1 expression.

## CONFLICT OF INTEREST

The authors declare no conflict of interest.

## References

[1] Boscher C , Nabi IR . Caveolin‐1: role in cell signaling. Adv Exp Med Biol. 2012;729:29‐50. 22411312 10.1007/978-1-4614-1222-9_3

[2] Bender FC , Reymond MA , Bron C , Quest AF . Caveolin‐1 levels are down‐regulated in human colon tumors, and ectopic expression of caveolin‐1 in colon carcinoma cell lines reduces cell tumorigenicity. Cancer Res. 2000;60:5870‐5878. 11059785

[3] Engelman JA , Wykoff CC , Yasuhara S , Song KS , Okamoto T , Lisanti MP . Recombinant expression of caveolin‐1 in oncogenically transformed cells abrogates anchorage‐independent growth. J Biol Chem. 1997;272:16374‐16381. 9195944 10.1074/jbc.272.26.16374

[4] Hino M , Doihara H , Kobayashi K , Aoe M , Shimizu N . Caveolin‐1 as tumor suppressor gene in breast cancer. Surg Today. 2003;33:486‐490. 14506991 10.1007/s10595-002-2538-4

[5] Racine C , Belanger M , Hirabayashi H , Boucher M , Chakir J , Couet J . Reduction of caveolin 1 gene expression in lung carcinoma cell lines. Biochem Biophys Res Commun. 1999;255:580‐586. 10049753 10.1006/bbrc.1999.0236

[6] Williams TM , Lisanti MP . Caveolin‐1 in oncogenic transformation, cancer, and metastasis. Am J Physiol Cell Physiol. 2005;288:C494‐C506. 15692148 10.1152/ajpcell.00458.2004

[7] Nethe M , Hordijk PL . A model for phospho‐caveolin‐1‐driven turnover of focal adhesions. Cell Adh Migr. 2011;5:59‐64. 20948305 10.4161/cam.5.1.13702 PMC3038100

[8] Yang B , Radel C , Hughes D , Kelemen S , Rizzo V . p190 RhoGTPase‐activating protein links the beta1 integrin/caveolin‐1 mechanosignaling complex to RhoA and actin remodeling. Arterioscler Thromb Vasc Biol. 2011;31:376‐383. 21051664 10.1161/ATVBAHA.110.217794 PMC3140163

[9] Lin HH , Lin HK , Lin IH , et al. Mechanical phenotype of cancer cells: cell softening and loss of stiffness sensing. Oncotarget. 2015;6:20946‐20958. 26189182 10.18632/oncotarget.4173 PMC4673241

[10] Wodarz A , Nathke I . Cell polarity in development and cancer. Nat Cell Biol. 2007;9:1016‐1024. 17762893 10.1038/ncb433

[11] Borghi N , Sorokina M , Shcherbakova OG , et al. E‐cadherin is under constitutive actomyosin‐generated tension that is increased at cell‐cell contacts upon externally applied stretch. Proc Natl Acad Sci U S A. 2012;109:12568‐12573. 22802638 10.1073/pnas.1204390109 PMC3411997

[12] Oakley C , Brunette DM . Response of single, pairs, and clusters of epithelial cells to substratum topography. Biochem Cell Biol. 1995;73:473‐489. 8703418 10.1139/o95-053

[13] Twiss F , de Rooij J . Cadherin mechanotransduction in tissue remodeling. Cell Mol Life Sci. 2013;70:4101‐4116. 23563964 10.1007/s00018-013-1329-x PMC11113614

[14] Galbiati F , Volonte D , Brown AM , et al. Caveolin‐1 expression inhibits Wnt/beta‐catenin/Lef‐1 signaling by recruiting beta‐catenin to caveolae membrane domains. J Biol Chem 2000;275:23368‐23377. 10816572 10.1074/jbc.M002020200

[15] Hackett TL , de Bruin HG , Shaheen F , et al. Caveolin‐1 controls airway epithelial barrier function: implications for asthma. Am J Respir Cell Mol Biol. 2013;49:662‐671. 23742006 10.1165/rcmb.2013-0124OC

[16] Antonyak MA , Cerione RA . Microvesicles as mediators of intercellular communication in cancer. Methods Mol Biol. 2014;1165:147‐173. 24839024 10.1007/978-1-4939-0856-1_11

[17] Greening DW , Gopal SK , Mathias RA , et al. Emerging roles of exosomes during epithelial‐mesenchymal transition and cancer progression. Semin Cell Dev Biol. 2015;40:60‐71. 25721809 10.1016/j.semcdb.2015.02.008

[18] Miller IV , Grunewald TG . Tumour‐derived exosomes: Tiny envelopes for big stories. Biol Cell. 2015;107:287‐305. 25923825 10.1111/boc.201400095

[19] Nazimek K , Bryniarski K , Santocki M , Ptak W . Exosomes as mediators of intercellular communication: clinical implications. Pol Arch Med Wewn. 2015;125:370‐380. 25978300 10.20452/pamw.2840

[20] Tauro BJ , Mathias RA , Greening DW , et al. Oncogenic H‐ras reprograms Madin‐Darby canine kidney (MDCK) cell‐derived exosomal proteins following epithelial‐mesenchymal transition. Mol Cell Proteomics. 2013;12:2148‐2159. 23645497 10.1074/mcp.M112.027086 PMC3734576

[21] Goetz JG , Joshi B , Lajoie P , et al. Concerted regulation of focal adhesion dynamics by galectin‐3 and tyrosine‐phosphorylated caveolin‐1. J Cell Biol. 2008;180:1261‐1275. 18347068 10.1083/jcb.200709019 PMC2290850

[22] Chen WC , Lin HH , Tang MJ . Regulation of proximal tubular cell differentiation and proliferation in primary culture by matrix stiffness and ECM components. Am J Physiol Renal Physiol. 2014;307:F695‐F707. 25056346 10.1152/ajprenal.00684.2013

[23] Yeh YC , Ling JY , Chen WC , Lin HH , Tang MJ . Mechanotransduction of matrix stiffness in regulation of focal adhesion size and number: reciprocal regulation of caveolin‐1 and beta1 integrin. Sci Rep. 2017;7:15008. 29118431 10.1038/s41598-017-14932-6 PMC5678369

[24] Chiou YW , Lin HK , Tang MJ , Lin HH , Yeh ML . The influence of physical and physiological cues on atomic force microscopy‐based cell stiffness assessment. PLoS ONE. 2013;8:e77384. 24194882 10.1371/journal.pone.0077384 PMC3806741

[25] Chen YS , Mathias RA , Mathivanan S , et al. Proteomics profiling of Madin‐Darby canine kidney plasma membranes reveals Wnt‐5a involvement during oncogenic H‐Ras/TGF‐beta‐mediated epithelial‐mesenchymal transition. Mol Cell Proteomics. 2011;10:001131. 20511395 10.1074/mcp.M110.001131 PMC3033669

[26] Chen Q , Takada R , Takada S . Loss of Porcupine impairs convergent extension during gastrulation in zebrafish. J Cell Sci. 2012;125:2224‐2234. 22357957 10.1242/jcs.098368

[27] Proffitt KD , Madan B , Ke Z , et al. Pharmacological inhibition of the Wnt acyltransferase PORCN prevents growth of WNT‐driven mammary cancer. Cancer Res. 2013;73:502‐507. 23188502 10.1158/0008-5472.CAN-12-2258

[28] Gujral TS , Chan M , Peshkin L , Sorger PK , Kirschner MW , MacBeath G . A noncanonical Frizzled2 pathway regulates epithelial‐mesenchymal transition and metastasis. Cell. 2014;159:844‐856. 25417160 10.1016/j.cell.2014.10.032 PMC4243058

[29] Razani B , Altschuler Y , Zhu L , Pestell RG , Mostov KE , Lisanti MP . Caveolin‐1 expression is down‐regulated in cells transformed by the human papilloma virus in a p53‐dependent manner. Replacement of caveolin‐1 expression suppresses HPV‐mediated cell transformation. Biochemistry. 2000;39:13916‐13924. 11076533 10.1021/bi001489b

[30] Kikuchi A , Yamamoto H . Tumor formation due to abnormalities in the beta‐catenin‐independent pathway of Wnt signaling. Cancer Sci. 2008;99:202‐208. 18271916 10.1111/j.1349-7006.2007.00675.x PMC11159738

[31] Gross JC , Chaudhary V , Bartscherer K , Boutros M . Active Wnt proteins are secreted on exosomes. Nat Cell Biol. 2012;14:1036‐1045. 22983114 10.1038/ncb2574

[32] Zhang L , Wrana JL . The emerging role of exosomes in Wnt secretion and transport. Curr Opin Genet Dev. 2014;27:14‐19. 24791688 10.1016/j.gde.2014.03.006

[33] Harada T , Yamamoto H , Kishida S , et al. Wnt5b‐associated exosomes promote cancer cell migration and proliferation. Cancer Sci. 2017;108:42‐52. 27762090 10.1111/cas.13109 PMC5276837

[34] Luga V , Zhang L , Viloria‐Petit AM , et al. Exosomes mediate stromal mobilization of autocrine Wnt‐PCP signaling in breast cancer cell migration. Cell. 2012;151:1542‐1556. 23260141 10.1016/j.cell.2012.11.024

[35] Kumawat K , Gosens R . WNT‐5A: signaling and functions in health and disease. Cell Mol Life Sci. 2016;73:567‐587. 26514730 10.1007/s00018-015-2076-y PMC4713724

[36] Asano T , Yamada S , Fuchs BC , et al. Clinical implication of Frizzled 2 expression and its association with epithelial‐to‐mesenchymal transition in hepatocellular carcinoma. Int J Oncol. 2017;50:1647‐1654. 28350091 10.3892/ijo.2017.3937

[37] Bian Y , Chang X , Liao Y , et al. Promotion of epithelial‐mesenchymal transition by Frizzled2 is involved in the metastasis of endometrial cancer. Oncol Rep. 2016;36:803‐810. 27373314 10.3892/or.2016.4885

[38] Zhang E , Li Z , Xu Z , Duan W , Sun C , Lu L . Frizzled2 mediates the migration and invasion of human oral squamous cell carcinoma cells through the regulation of the signal transducer and activator of transcription‐3 signaling pathway. Oncol Rep. 2015;34:3061‐3067. 26398330 10.3892/or.2015.4285

[39] Asem MS , Buechler S , Wates RB , Miller DL , Stack MS . Wnt5a signaling in cancer. Cancers (Basel). 2016;8:79. 10.3390/cancers8090079 PMC5040981 27571105

[40] Quest AF , Gutierrez‐Pajares JL , Torres VA . Caveolin‐1: an ambiguous partner in cell signalling and cancer. J Cell Mol Med. 2008;12:1130‐1150. 18400052 10.1111/j.1582-4934.2008.00331.x PMC3865655

[41] Nethe M , Anthony EC , Fernandez‐Borja M , et al. Focal‐adhesion targeting links caveolin‐1 to a Rac1‐degradation pathway. J Cell Sci. 2010;123:1948‐1958. 20460433 10.1242/jcs.062919

[42] Felley‐Bosco E , Bender FC , Courjault‐Gautier F , Bron C , Quest AF . Caveolin‐1 down‐regulates inducible nitric oxide synthase via the proteasome pathway in human colon carcinoma cells. Proc Natl Acad Sci U S A. 2000;97:14334‐14339. 11114180 10.1073/pnas.250406797 PMC18919

[43] Chen SF , Wu CH , Lee YM , et al. Caveolin‐1 interacts with Derlin‐1 and promotes ubiquitination and degradation of cyclooxygenase‐2 via collaboration with p97 complex. J Biol Chem. 2013;288:33462‐33469. 24089527 10.1074/jbc.M113.521799 PMC3829191

[44] Liu P , Rudick M , Anderson RG . Multiple functions of caveolin‐1. J Biol Chem. 2002;277:41295‐41298. 12189159 10.1074/jbc.R200020200

[45] Parton RG , Simons K . The multiple faces of caveolae. Nat Rev Mol Cell Biol. 2007;8:185‐194. 17318224 10.1038/nrm2122

[46] Doroudi M , Olivares‐Navarrete R , Hyzy SL , Boyan BD , Schwartz Z . Signaling components of the 1alpha,25(OH)2D3‐dependent Pdia3 receptor complex are required for Wnt5a calcium‐dependent signaling. Biochim Biophys Acta. 2014;1843:2365‐2375. 24946135 10.1016/j.bbamcr.2014.06.006 PMC4287416

[47] Brinton LT , Sloane HS , Kester M , Kelly KA . Formation and role of exosomes in cancer. Cellular and molecular life sciences: CMLS. 2015;72:659‐671. 25336151 10.1007/s00018-014-1764-3 PMC5489338

[48] Zhang X , Yuan X , Shi H , Wu L , Qian H , Xu W . Exosomes in cancer: small particle, big player. J Hematol Oncol. 2015;8:83. 26156517 10.1186/s13045-015-0181-x PMC4496882

[49] Bissell MJ , Hines WC . Why don't we get more cancer? A proposed role of the microenvironment in restraining cancer progression. Nat Med. 2011;17:320‐329. 21383745 10.1038/nm.2328 PMC3569482

[50] Sieweke MH , Bissell MJ . The tumor‐promoting effect of wounding: a possible role for TGF‐beta‐induced stromal alterations. Crit Rev Oncog. 1994;5:297‐311. 7849089 10.1615/critrevoncog.v5.i2-3.90

[51] Williams TM , Sotgia F , Lee H , et al. Stromal and epithelial caveolin‐1 both confer a protective effect against mammary hyperplasia and tumorigenesis: Caveolin‐1 antagonizes cyclin D1 function in mammary epithelial cells. Am J Pathol. 2006;169:1784‐1801. 17071600 10.2353/ajpath.2006.060590 PMC1780215

[52] Svensson KJ , Christianson HC , Wittrup A , et al. Exosome uptake depends on ERK1/2‐heat shock protein 27 signaling and lipid Raft‐mediated endocytosis negatively regulated by caveolin‐1. J Biol Chem. 2013;288:17713‐17724. 23653359 10.1074/jbc.M112.445403 PMC3682571

[53] Zou H , Stoppani E , Volonte D , Galbiati F . Caveolin‐1, cellular senescence and age‐related diseases. Mech Ageing Dev. 2011;132:533‐542. 22100852 10.1016/j.mad.2011.11.001 PMC3243775

