# Supplementary material for: Caveolin‐1 down‐regulation is required for Wnt5a‐Frizzled 2 signalling in Ha‐RasV12‐induced cell transformation
Source: J Cell Mol Med. 2018 Mar 4;22(5):2631–43. doi: 10.1111/jcmm.13531 (PMC5908114; doi:10.1111/jcmm.13531)

Supplementary Figures and Figure legends

Fig. S1 Overexpression of Cav1 abolished Ha-Ras^{V12}-induced disorganization of cellular junctions in MK4 cells. (**A**) Representative immunoblots for E-cadherin, vinculin, α-catenin, β-catenin, γ-catenin, and ZO-1 in MK4 cells treated with or without IPTG (5 mM) for 24 h. β-actin served as loading control. (**B**) Representative confocal images for β-catenin, E-cadherin (E-cad), ZO-1, caveolin-1, and ZO-1 in MK4 cells treated with or without IPTG for 24 h. Nucleus: blue. Scale bar=10 μm. Representative confocal images for (**C**) E-cadherin, (**D**) β-catenin, and (**E**) claudin-1 of MK4+Cav1 cells treated with or without IPTG for 24 h. Nucleus: blue. Scale bar = 10 μm.

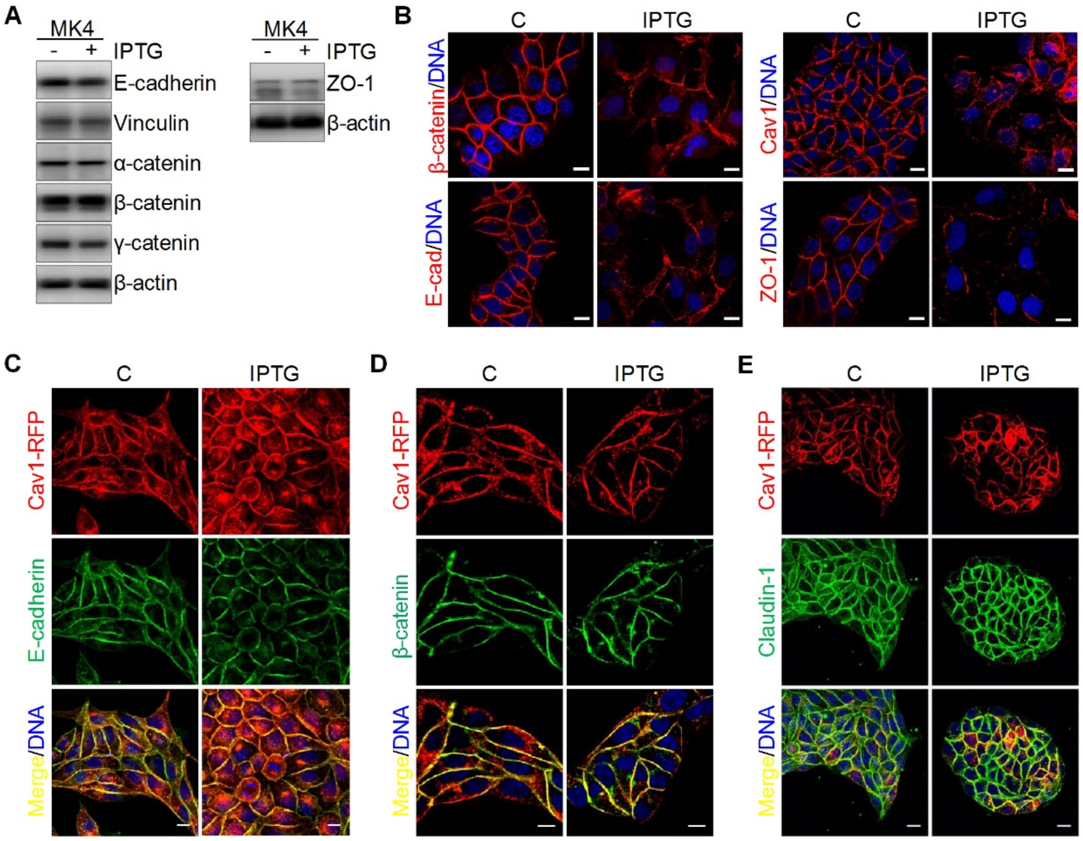

Fig. S2 shRNA silencing of Cav1 does not induce cell scattering or cell softening in both MK4 cells and MDCK cells. MK4 cells and MDCK cells were stably transfected with non-targeting control shRNA (MDCK/shNC) or Cav1 shRNA (MDCK/shCav1, clones #1 and #2). (**A**) and (**B**) Representative immunoblots for Cav1 of the indicated cells. β-actin served as loading control. (**C**) and (**D**) AFM indentation results for cell stiffness of the indicated cells plated on culture dish for 24 h. (**E**) and (**F**) Transwell migration ability of indicated cells. (**G**) and (**H**) The measurement for cell area of the indicated cells plated on culture dish for 24 h. (**I**) and (**J**) Representative confocal images for F-actin (Alexa-594 phalloidin, red) and Cav1 (green) of the indicated cells. Nucleus: blue. Scale bar = 20 μm. Error bars indicate mean ± S.E.M. from two independent experiments; ***P<0.001.

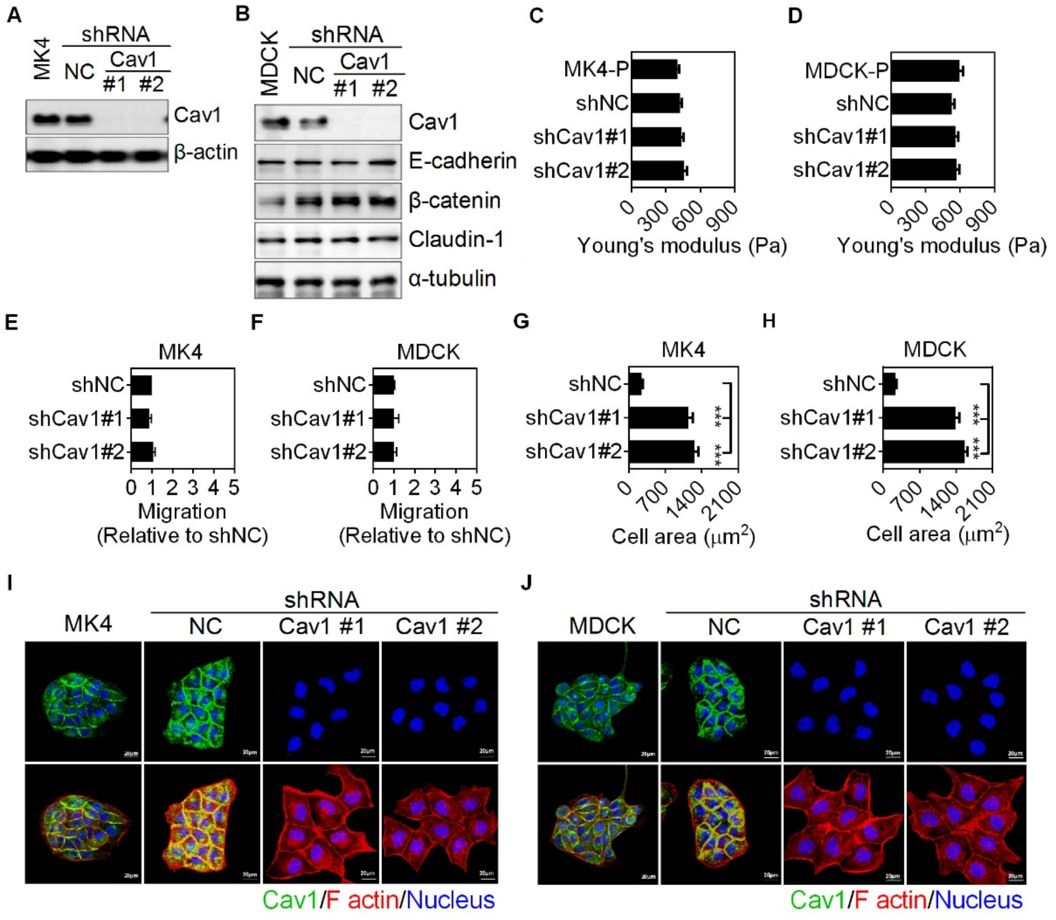

Fig. S3 Silencing Cav1 expression results in weakened and discontinuous cell junction and promotes the responsiveness of MDCK/shCav1 cells to Ha-Ras^{V12}-activated medium. MDCK cells were stably transfected with non-targeting control shRNA (MDCK/shNC) or Cav1 shRNA (MDCK/shCav1, clones #1 and #2). (**A**) Representative confocal images for E-cadherin, β-catenin, and ZO-1 of the indicated cells. Nucleus: blue. Scale bar = 20 μm. (**B**) Representative confocal images for β-catenin, E-cadherin (E-cad), claudin-1, and ZO-1 of indicated cells treated with medium conditioned by MK4 cells (MK4-CM) or MK4 cells treated with IPTG (MK4+I-CM) for 24 h. Scale bar=10 μm.

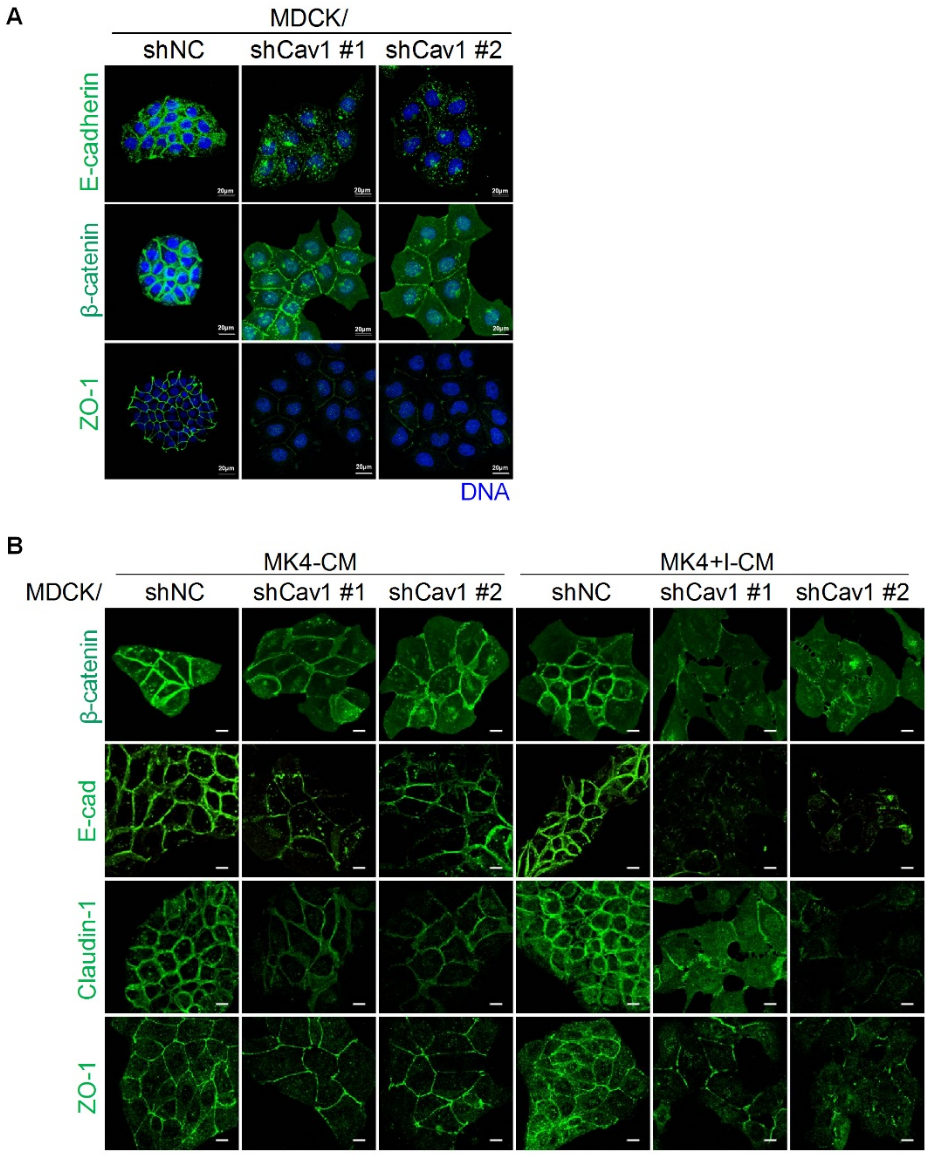

Fig. S4 The cellular transformation ability of MK4+I-CM is mediated by exosomes (EXs), which are heat labile. MDCK cells were stably transfected with shNC (MDCK/shNC) or shCav1 (MDCK/shCav1, clones #1 and #2). (**A**) Phase contrast images, (**B**) AFM indentation results (n=2), (**C**) Transwell migration ability and matrigel invasion ability of the indicated cells treated with MK4+I-CM or heated MK4+I-CM for 24 h (n=2). (**D**) Phase contrast images of the indicated cells treated with MK4^{Ras}-CM, EXs depleted MK4+I-CM, EXs replenished/depleted MK4+I-CM, and Wnt5a supplemented/EXs depleted MK4+I-CM. (**E**) Phase contrast images of the indicated cells treated with various concentrations of EXs isolated from MK4 cells treated with IPTG (MK4+I-EXs) or heated MK4+I-EXs (300 μg/ml). Scale bars = 100 μm. Error bars indicate mean ± S.E.M.; ***P<0.001.

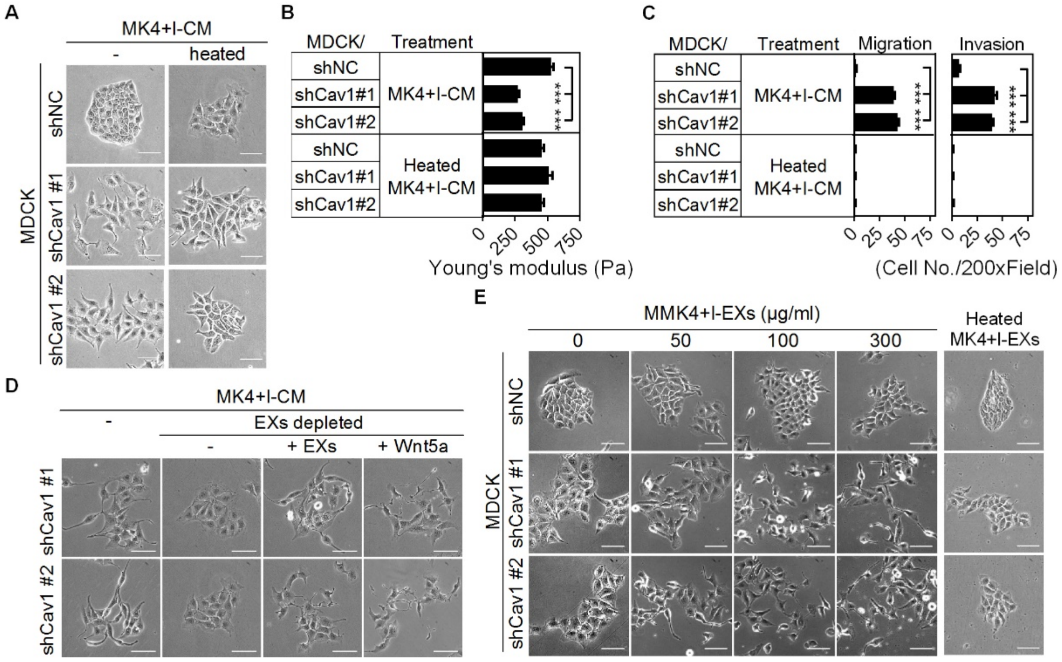

Fig. S5 Wnt5a is responsible for MK4+I-CM-induced cell scattering of MDCK/shCav1 cells. (**A**) Phase contrast images of MDCK/shNC and MDCK/shCav1 (clones #1 and #2) treated with various concentrations of Wnt5a. (**B**) Western blot results of MK4 cells treated with or without IPTG in the absence or the presence of various concentration of C59. The protein levels of Wnt5a were analyzed. α-tubulin was used as an internal control. (**C**) Phase contrast images of MK4 cells treated with or without IPTG in the absence or the presence of C59 (200 nM). (**D**) Phase contrast images of MDCK/shCav1 cells (clones #1 and #2) treated with medium conditioned by MK4 cells treated with or without 5 mM IPTG in the absence or the presence of C59 (200 nM). (**E**) Western blot results of MK4 cells treated with IPTG and non-targeting control siRNA (siNC) or various concentration of siWnt5a. The protein levels of Wnt5a were analyzed. α-tubulin was used as an internal control. Scale bars = 100 μm.

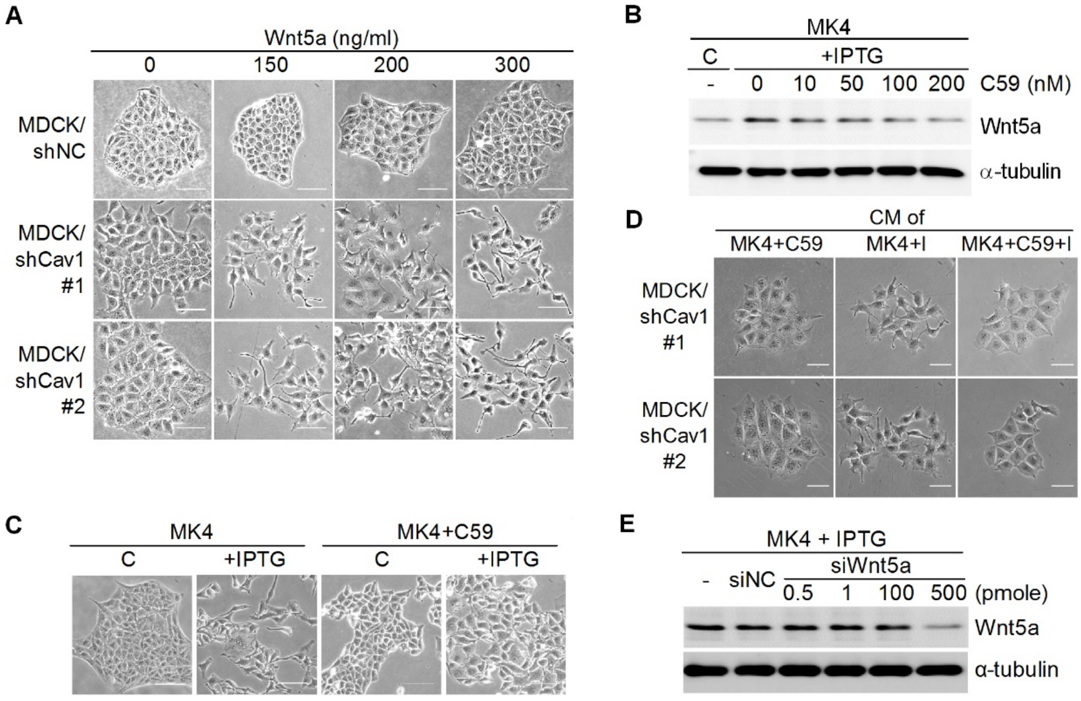

Fig. S6 WP1066 (Stat3 inhibitor) diminished Ha-Ras^{V12}- or Wnt5a-enhanced cellular migration in (A) MK4 cells or (B) MDCK/shCav1 cells (clone #1 and #2), respectively. Transwell migration of indicated cells treated with or without IPTG or Wnt5a in the presence of DMSO or WP1066 for 24 h.

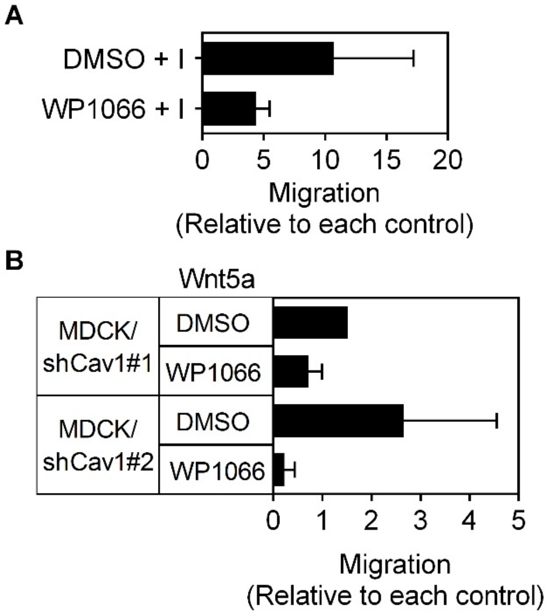

Fig. S7 RT-PCR results for Fzd2, Fzd5, and Fzd8 expression of MK4 cells treated with or without IPTG for 24 h. β actin was used as an internal control.

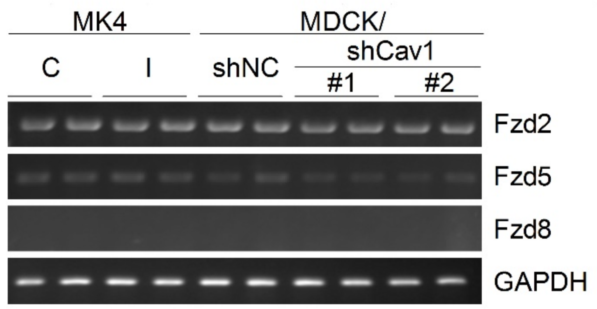

Fig. S8 Inhibition of protein synthesis by cycloheximide (CHX) transiently increased the protein levels of Fzd2 within 4 h only in Cav1-expressing cells, including MK4 cells and MDCK/shNC cells. (A) Representative immunoblots for Fzd2 and Cav1 in MK4 cells treated for 24 h with or without IPTG before the addition of CHX (20 μg/ml) for the indicated times. GAPDH served as an internal control. (B) The quantitative result of Fzd2 from (A) and two other experiments (n=3). GAPDH-normalized data in each conditions was compared with 0 h. (C) Representative immunoblots for Fzd2 and Cav1 in MDCK/shNC cells and MDCK/shCav1 cells (clone #1 and #2) treated CHX (20 μg/ml) for the indicated times. GAPDH served as an internal control. (D) The quantitative result of Fzd2 from (C) and two other experiments (n=3). GAPDH-normalized data in each conditions was compared with 0 h. Error bars indicate mean ± S.E.M.; **P < 0.01.

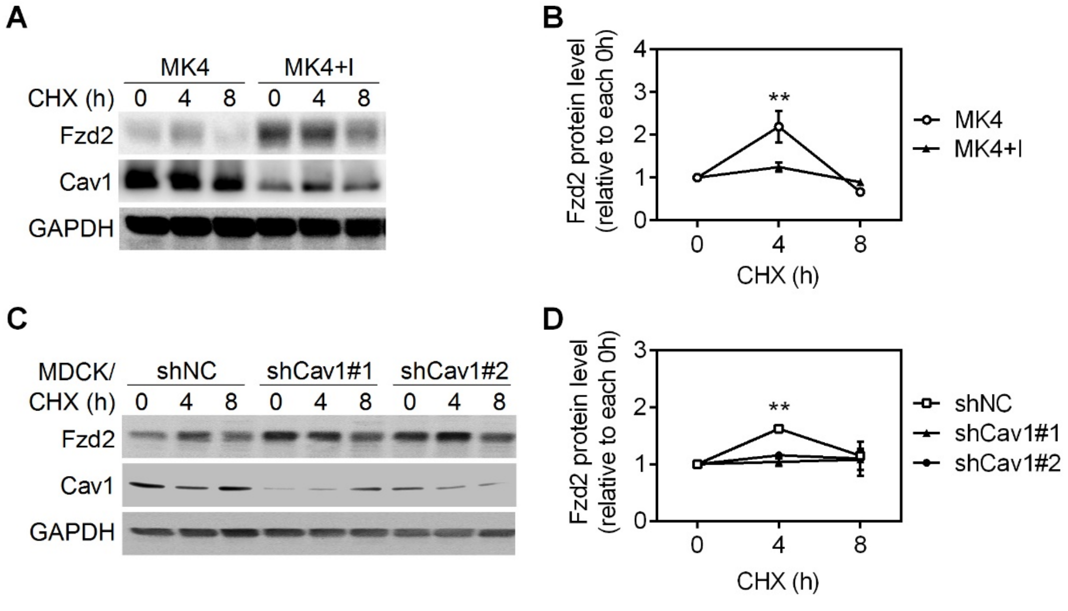

Supplement: Supplementary file 1 [file JCMM-22-2631-s001.pdf]
